# Research on Logistics Distribution Center Location Based on Hybrid Beetle Antennae Search and Rain Algorithm

**DOI:** 10.3390/biomimetics7040194

**Published:** 2022-11-07

**Authors:** Zhimin Mei, Xuexin Chi, Rui Chi

**Affiliations:** 1 Wuchang Institute of Technology Robotics Application Institute, Wuchang Institute of Technology, Wuhan 430070, China; 2 School of Electrical and Automation Engineering, East China Jiaotong University, Nanchang 330013, China

**Keywords:** logistics location, optimization, beetle antennae search algorithm, rain algorithm, BRA

## Abstract

The location of logistics distribution centers is a crucial issue in modern logistics distribution systems. In order to obtain a more reasonable solution, an effective optimization algorithm is essential. This paper proposes a new hybrid method, named the beetle antennae search–rain algorithm (BRA), for the problem of logistics distribution centers’ location. The innovation of the BRA is embodied in three aspects. Firstly, the beetle antennae search (BAS) algorithm is embedded into the rain algorithm (RA); thus, the BAS is improved from an individual search to a swarm intelligent search and the global search ability is improved. Secondly, the search direction strategy of the BAS algorithm is incorporated into the RA, which can improve response speed while ensuring optimization performance. Finally, the search precision is improved by the mechanism of eliminating the inferior solution and generating a new solution. The BRA is tested on 10 benchmark functions and applied to solve the logistics distribution centers’ location problem. The performance of the BRA is compared to that of several classical heuristics by using relevant evaluation indexes and dynamic optimization convergence graphs. Experimental results show that the BRA outperforms the BAS algorithm, the RA and some other classic heuristics. It is also revealed that the BRA is an effective and competitive algorithm for logistics distribution centers’ location.

## 1. Introduction

The location of logistics distribution centers is a planning process to select a suitable location to establish a distribution center in a region, including several demand points [1]. The logistics distribution center is an important part of the logistics distribution system, which decides the whole function of the logistics distribution system [2]. The location decision of logistics distribution centers is not only related to the future operating income and the expenditure of the logistics distribution center, as well as the level of service to customers, but it is also related to the rationalization of the whole logistics system [3]. Considering the influence of complex conditions, such as transportation conditions, customer demand and the distribution of goods, the scientific selection of the location and the quantity of logistics distribution centers can effectively save the cost of investment and ensure the efficient operation of the whole logistics system, which is of great significance to the management of the whole logistics chain [4,5]. The location of logistics distribution centers is a non-convex mathematical model with complex constraints, which belongs to NP-hard nonlinear programming [6]. There are many classical methods to solve this mathematical model, such as the gravity method [7], the bilevel programming method [8], the Branch and Bound method [9] and so on. With the rapid development of the logistics market, the scale of the logistics distribution system is becoming larger and larger, and it is more and more difficult to solve this mathematical model by traditional methods [10]. Therefore, it is necessary to explore efficient algorithms to solve this problem. In recent years, heuristic algorithms have been widely used in complex optimization problems, and it also provides a new way to solve the logistics distribution centers’ location problem. For example, Wang, Y. and Ma, X.L. et al. presented a hybrid particle-swarm optimization–genetic algorithm for a two-echelon logistics distribution region-partitioning problem [11]; Cui, H.X. and Qiu, J.L. et al. used an adaptive genetic algorithm to solve route optimization in township logistics distribution based on customer satisfaction [12]; Enrique, D. and José, M. proposed a neural model for the p-median problem, which is also one of the most popular and well-known facility location problems [13]; Thongdee, T. and Pitakaso, R. presented a differential evolution algorithms to solve a multi-objective, source and stage location–allocation problem [14]; Jha, A. and Somani, K. et al. used a modified adaptive differential evolution algorithm to minimize the transportation cost of a joint inventory location model [15]; Wang, L. and Qu, H. et al. adopted an effective hybrid self-adapting deferential evolution algorithm for the joint replenishment and location–inventory problem in a tree-level, supply-level supply chain [16]; Hua, X. and Hu, X. et al. presented an adaptive particle-swarm algorithm for the location of logistics distribution centers [17]; Qi, C.M. adopted an improved discrete particle-swarm optimization in the logistics distribution routing problem [18]; Liu, H.J. proposed an ant colony algorithm for cloud-computing adaptive task scheduling based on an ant colony algorithm [19]; Teschemacher, U. and Reinhart, G. presented ant colony optimization algorithms to solve and enable dynamic milk-run logistics [20]; and so on. The application of heuristics to the logistics distribution centers’ location problem and the improvement of original algorithms to enhance the performance of solving the logistics distribution centers’ location problem by scholars have a profound impact on the development of the logistics distribution centers’ location.

Although these improvements have been made with the use of heuristic algorithm to solve the logistics distribution centers’ location problem, it is worth further study because of its multiple constraints and complexity. Therefore, new algorithms to solve this problem have attracted more and more attention in recent years. The beetle antennae search (BAS) algorithm is an excellent bionic algorithm designed according to the feeding characteristics of beetles [21,22]. It has the characteristics of good generality and being easy to integrate into other algorithms, and it has been successfully applied in many fields, such as image processing [23], predictive controls [24], neural networks [25] and so on. The rain algorithm (RA) is a kind of swarm intelligence optimization algorithm based on the raindrop phenomenon in nature. It has a good fine-tuning ability to avoid the algorithm falling into local optimization, and it can maintain good diversity of the population [26]. In this paper, a hybrid algorithm named BRA is proposed based on the analysis of the disadvantages and advantages of the BAS algorithm and the RA, and it is used to solve the location problem of the logistics distribution center. The experimental results demonstrate the effectiveness of the hybrid algorithm.

## 2. Logistics Distribution Center Location Model

This paper studies how to select some locations for logistics distribution centers in a limited number of locations, and how to provide transportation for all locations so as to minimize the total cost. Therefore, the following assumptions were made:

- The logistics distribution center needs to satisfy the demand of all the locations.
- Each demand location has and can only be supplied by one logistics distribution center.
- The product of $M_{d}$ and $N_{d}$ is the smallest, where $M_{d}$ is the demand of each location and $N_{d}$ is the distance to the nearest distribution center.
- Other costs are not considered in this paper.

The logistics distribution center location model based on the above assumptions is as follows [2,27]:

(1) $$minF=\sum_{i\in I}\sum_{j\in M_{i}}h_{i}d_{ij}z_{ij},$$

(2) $$s.t.\sum_{j\in M_{i}}z_{ij}=1,i\in I,$$

(3) $$z_{ij}\leq e_{j},i\leq I,j\leq M_{i},$$

(4) $$\sum_{j\in M_{i}}e_{j}=r,$$

(5) $$z_{ij},e_{j}\in \left\{0,1\right\},i\in I,j\in M_{i},$$

(6) $$d_{ij}\leq k,$$

where the objective function minF represents the minimum product of the demand quantity and the distance from the selected logistics distribution center j to the location i distributed by it; k represents the maximum distance from the selected logistics distribution center j to the location i to be distributed by it; $M_{i}$ represents the collection of other logistics distribution centers whose distance from other locations to the distribution center is less than k; $h_{i}$ is the demand for position i; and $d_{ij}$ represents the distance between location i and the nearest distribution center j. The distribution area shall be within k.

Equations (2)–(6) represent the constraints of the model. Equation (2) guarantees that each demand point can only be served by one distribution center. Equation (3) indicates that there will be no customers at locations that do not have distribution centers. Equation (4) specifies that the number of distribution centers selected is r. Equation (5) indicates that $z_{ij}$ and $e_{j}$ are the 0–1 variables. Equation (6) ensures that the demand point is within the range of the distribution center.

## 3. The Hybrid BRA

### 3.1. The Original BAS Algorithm

The BAS algorithm is an intelligent optimization algorithm based on the foraging behavior of beetles, proposed by researchers Xiangyuan Jiang and Shuai Li in 2017. The beetle has two long antennae. During the process of feeding, if the left antennae receives a higher smell intensity than the right, then the beetle will, in its next step, fly to the left. Otherwise, it will fly to the right. Based on this simple principle, the beetle can effectively find food. The original BAS algorithm consists of the following steps:

- Step 1: Build a search model that describes a random direction of the beetle’s search,
  (7) $$\vec{b}=\frac{rands(D,1)}{\left\|rands(D,1)\right\|},$$
  where rands( ) is a random function and D is the dimension of solution space.
- Step 2: Create the space coordinates of beetle’s left and right antennae,
  (8) $$\left\{ \begin{array}{l} x_{rt}=x^{t}+d_{0}\times \vec{b}/2\\ x_{lt}=x^{t}-d_{0}\times \vec{b}/2 \end{array}\right.,$$
  where $x_{lt}$ and $x_{rt}$ indicate the position of the left and right antennae of the longicorn beetle on the t iteration, respectively; $x^{t}$ indicates the position of the centroid of the longicorn beetle on the t iteration; and $d_{0}$ indicates the distance between the left and right antennae.
- Step 3: The fitness function is used to judge the intensity of food smell received by the left and right antennae, that is, the values of $f(x_{l})$ and $f(x_{r})$. f( ) denotes the fitness function.
- Step 4: Update the position of the longhorn beetle based on Equation (9),
  (9) $$x^{t}=x^{t-1}+\delta^{t}\times \vec{b}\times sign(f(x_{r})-f(x_{l})),$$
  where $\delta^{t}$ denotes the step size factor at the t iteration and sign( ) is a symbolic function.
- Step 5: Determine whether the current solution meets the stop condition. If this condition is satisfied, stop the execution. Otherwise, jump to Step 2 and execute the loop.

### 3.2. The Original RA

The RA is a swarm intelligence search algorithm proposed by researchers Chi Rui and Chi Xuexin, who were inspired by the natural raindrop phenomenon in 2022. This algorithm has three parts: generate the initial raindrops; split and recombine the raindrops; and flow to the lowest position. The steps of the RA are as follows:

- Step 1: A random number of raindrops are produced in dark clouds, as shown in Equation (10):
  (10) $${\vec{x}}_{i}^{initial}={\vec{x}}^{min}+rand(0,1)\times ({\vec{x}}^{max}-{\vec{x}}^{min}),i=1,2,\ldots ,N_{P},$$
  where rand(0,1) is a random number with uniform distribution. ${\vec{x}}^{min}$ and ${\vec{x}}^{max}$ are the upper and lower limits of the solution space, respectively.
- Step 2: The raindrop falls on the ground and splits into many small raindrops, as shown in Equation (11):
  (11) $$\left\{ \begin{array}{l} {\vec{u}}_{j}{}^{t}={\vec{x}}_{i}{}^{t}+rand(-1,1)\times R^{t}\;,j=1,2,\ldots ,N_{S}\\ R^{t}=R^{{}_{max}}-\frac{t}{t^{{}_{max}}}\times (R^{{}_{max}}-R^{{}_{min}}) \end{array}\right.,$$
  where ${\vec{u}}_{j}{}^{t}$ is the position of the split little raindrop. rand(−1,1) is a group of uniformly distributed D-dimensional random numbers. $R^{t}$ represents the coverage radius of a raindrop that splits into small raindrops. $R^{{}_{max}}$ and $R^{{}_{min}}$ represent the maximum and minimum values of $R^{t}$, respectively.
- Step 3: These split small raindrops are recombined into large raindrops based on Equation (12):
  (12) $${\vec{x}}_{i}^{t}=\frac{1}{N_{S}}\cdot \sum_{j=1}^{N_{S}}{\vec{u}}_{j}^{t},$$
  where $N_{S}$ represents the number of the split small raindrops.
- Step 4: The weight $\omega_{i}^{t}$ of the raindrops is calculate and the position of the current individual ${\vec{x}}_{i}^{t}$ is updated according to the range of $\omega_{i}^{t}$, as shown in Equation (13):
  (13) $$\begin{array}{l} {\vec{x}}_{i}^{t+1}=(1-\omega_{i}^{t})\times rand(-1,1)\times V_{Pi}^{t}\times {\vec{x}}_{i}^{t}+\omega_{i}^{t}\times rand(-1,1)\times V_{Gi}^{t}\times {\vec{x}}_{best}^{t},\;\omega_{i}^{t}\in [\omega^{min},\omega^{max}]\\ {\vec{x}}_{i}^{t+1}={\vec{x}}^{min}+rand(0,1)\times ({\vec{x}}^{max}-{\vec{x}}^{min}),\;otherwise\\ \left\{ \begin{array}{l} \omega_{i}^{t}=\frac{f^{{}_{max}}-f({\vec{x}}_{i}^{t})}{f^{{}_{max}}-f^{{}_{min}}}\\ V_{Pi}^{t}=V_{P}{}^{{}_{max}}-\frac{t}{t^{{}_{max}}}\times (V_{P}{}^{{}_{max}}-V_{P}{}^{{}_{min}})\\ V_{Gi}^{t}=V_{G}{}^{{}_{max}}-\frac{t}{t^{{}_{max}}}\times (V_{G}{}^{{}_{max}}-V_{G}{}^{{}_{min}}) \end{array}\right. \end{array}$$
  where rand(−1,1) and rand(0,1) are random numbers that conform to the normal distribution; $V_{Pi}^{t}$ is the contraction factor of the current raindrop ${\vec{x}}_{i}^{t}$; $V_{P}{}^{{}_{max}}$ and $V_{P}{}^{{}_{min}}$ are the upper and lower limits of $V_{Pi}^{t}$, respectively; $V_{Gi}^{t}$ is the search factor of the optimal raindrop $V_{Gi}^{t}$; and $V_{Pi}^{t}$ and $V_{Gi}^{t}$ are the upper and lower limits of $V_{Gi}^{t}$, respectively. $V_{Gi}^{t}$ and $V_{Gi}^{t}$ decrease linearly.
- Step 5: The best raindrop is selected, it is determined whether the end condition is satisfied. If it is satisfied, the execution is stopped. Otherwise, the loop is executed after jumping to Step 2.

### 3.3. The Hybrid BRA Procedure

The BAS algorithm is an individual search behavior of a beetle in a solution space. It has a poor global search ability and easily falls into the local optimum. Even if it reaches near the global optimal solution, its convergence precision is lower than that of the swarm search algorithm. The RA is a kind of swarm intelligence algorithm, and its search strategy and BAS algorithm are close to each other; therefore, this paper embedded the BAS algorithm into the RA, proposing a hybridization called the BRA. This algorithm improves the location update equation from two aspects:

- In the BAS algorithm, the search direction of the beetle is determined by the fitness values of the left and right antennae. In the RA, the individual ${\vec{x}}_{i}^{t}$ with $\omega_{i}^{t}\in [\omega^{min},\omega^{max}]$ approaches the optimal position ${\vec{x}}_{best}^{t}$ of the current generation as the number of iterations increases. From Equation (13), it can be seen that the search direction of ${\vec{x}}_{i}^{t}$ is random. In order to better guide the search direction of ${\vec{x}}_{i}^{t}$, the search direction strategy of the BAS algorithm is introduced into the RA. It can be supposed that ${\vec{x}}_{best}^{t}$ represents the position of one beetle’s antennae and ${\vec{x}}_{i}^{t}$ is the position of another beetle’s antennae; therefore, the search direction of ${\vec{x}}_{i}^{t}$ can be determined by the position relation between ${\vec{x}}_{i}^{t}$ and ${\vec{x}}_{best}^{t}$, that is $\vec{b}\times sign({\vec{x}}_{i}^{t}-{\vec{x}}_{best}^{t})$, and the position update formula of individuals ${\vec{x}}_{i}^{t}$ of weight $\omega_{i}^{t}\in [\omega^{min},\omega^{max}]$ can be improved as follows:
  (14) $${\vec{x}}_{i}^{t+1}=(1-\omega_{i}^{t})\times rand(0,1)\times \vec{b}\times sign({\vec{x}}_{i}-{\vec{x}}_{i}^{best})\times V_{P}{}_{i}\times {\vec{x}}_{i}^{t}+\omega_{i}^{t}\times rand(-1,1)\times V_{G}{}_{i}\times {\vec{x}}_{best}^{t},$$
- In the RA, from Equation (13), it can be seen that the individuals ${\vec{x}}_{i}^{t}$ with $\omega_{i}^{t}\in [0,\omega^{min})$ and $\omega_{i}^{t}\in (\omega^{max},1]$ are discarded, and the same number of individuals are generated randomly in the solution space. The discarded solutions are the ones with a poor position or a lack of diversity near ${\vec{x}}_{best}^{t}$. The generated new solutions are randomly dispersed in the solution space which essentially increases the global search ability of the algorithm. Through the improvement of Equation (14), the global search ability of the individual ${\vec{x}}_{i}^{t}$ with $\omega_{i}^{t}\in [\omega^{min},\omega^{max}]$ is enhanced a lot. Therefore, in this paper, the individuals ${\vec{x}}_{i}^{t}$ with $\omega_{i}^{t}\in [0,\omega^{min})$ and $\omega_{i}^{t}\in (\omega^{max},1]$ are used to improve the local search ability. The same number of new solutions are randomly generated near ${\vec{x}}_{best}^{t}$ and the search direction $\vec{b}\times sign({\vec{x}}_{i}^{t}-{\vec{x}}_{best}^{t})$ is introduced, and the position update equation is improved as follows:
  (15) $${\vec{x}}_{i}^{t+1}={\vec{x}}_{best}^{t}+\vec{b}\times sign({\vec{x}}_{i}^{t}-{\vec{x}}_{best}^{t})\times rand(0,1)\;\cdot \;\nu ,$$
  where ν is the maximum radius raindrops near the lowest position and can be modified by the user based on actual needs.

**Figure 1:**
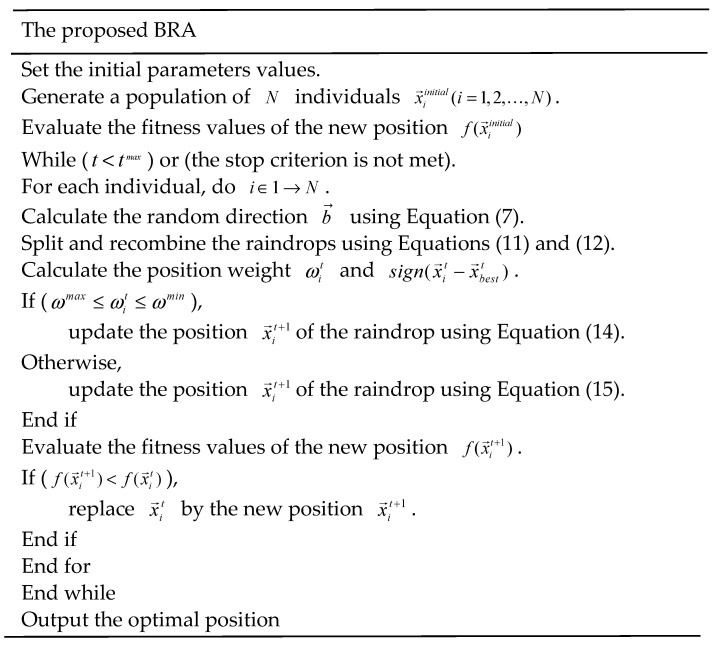
Pseudo-code of the proposed BRA.

From the above description, the proposed BRA can be described as shown in Figure 1.

## 4. Experimental Studies on Benchmark Functions

This section is divided by subheadings. It should provide a concise and precise description of the experimental results and their interpretation, as well as the experimental conclusions that can be drawn.

### 4.1. Benchmark Functions

In order to verify the optimization performance of the BRA, 10 classic benchmark function optimization problems were selected for testing [28,29]. The specific function expressions are shown in Table 1 (the dimensions are denoted by D). Among these benchmark functions, $F_{1}(x)$ to $F_{7}(x)$ are the unimodal functions, which can validate the convergence speed and search precision of the algorithm, and $F_{8}(x)$ to $F_{10}(x)$ are the multimodal functions with many local minima, which can verify the global search ability of the algorithm. These functions are widely used to verify the optimization performance of the algorithm.

### 4.2. Parameter Setting

In this paper, the BRA is compared with particle-swarm optimization (PSO), the genetic algorithm (GA), the bat-inspired algorithm (BA) [30], the BAS algorithm and the RA, comparing and analyzing their calculation results. For the above 10 classical function optimization problems, each algorithm runs independently 50 times under the same running environment (Intel Core i5-7200U 2.50 GHz processor, 4.0 GB memory and Windows 7 operating system with Matlab 2013b). The population size of the algorithms is denoted by N=20, and the maximum evolutionary algebra is denoted by $t^{{}_{max}}=2000$. The other parameters of these algorithms are set as follows:

- PSO: $c_{1}=c_{2}=2$, $w_{min}=0.4$, $w_{max}=0.9$;
- GA: pc=0.7, pm=0.3, mu=0.1;
- BA: A=0.25, r=1, $Q_{min}=0$, $Q_{max}=2$;
- BAS: eta=0.95, step=0.8, d=3;
- RA: $N_{S}=5$, $R^{max}=10$, $R^{min}=0.0005$, $V_{P}{}^{{}_{max}}=4$, $V_{P}{}^{{}_{min}}=0.0005$, $V_{G}{}^{{}_{max}}=2$, $V_{G}{}^{{}_{min}}=0.0005$;
- BRA: d=3, $N_{S}=5$, $\omega^{min}=0.2$, $\omega^{max}=0.8$, ν=5.

### 4.3. Numerical Results and Analysis

The results of PSO, the GA, the BA, the BAS algorithm, the RA and the BRA for the above 10 function optimization problems are calculated, and the best value (Best), worst value (Worst), mean value (Mean) and standard deviation value (Std) are taken as the evaluation indexes of the performance of these algorithms.

Table 2 shows the results of the six algorithms for 10 function optimization problems. The optimal results for each evaluation indexes are shown in bold. For the vast majority of these 10 function optimization problems, the BRA can obtain smaller results and perform better optimization performance.

The Best and Worst evaluation indexes represent the interval of the optimal value obtained by the algorithm in 50 independent runs. The smaller the value of these two evaluation indexes, the higher the accuracy of the algorithm. The Mean and Std evaluation indexes measure the stability of the algorithm in 50 independent runs. The closer the two evaluation indexes are, the higher the stability of the algorithm is. As can be seen from Table 2, for the unimodal function $F_{1}-F_{7}$, the Best and Worst values of the BRA are both minimum, and the Worst values of the BRA are smaller than the Best values of the other five algorithms. The results show that the BRA is much more accurate than the other five algorithms. The Mean and Std values of the BRA are of the same order of magnitude, which shows that the BRA can ensure high accuracy and good stability and robustness. For the multimodal function $F_{8}$ and $F_{9}$, the values of the four evaluation indexes for the BRA are all 0, which shows that the algorithm obtained the theoretical optimal value in 50 independent runs, and it can easily jump out of the local optimal value. It shows excellent global search ability, high precision and reliable stability. For the multimodal function $F_{10}$, although the BRA did not find the theoretical optimal value, but compared with the other five algorithms, the value of its four evaluation indexes is the smallest, and it shows excellent optimization performance. To sum up, the BRA, for the 10 benchmark functions in Table 1, can obtain relatively good results.

In order to directly compare the performance of PSO, the GA, the BA, the BAS algorithm, the RA and the BRA, the following convergence curves of 10 typical function optimization problems are given. The vertical axis of the graph represents the optimal value obtained by each generation of the algorithm (for ease of comparison, the logarithm of the optimal function value obtained is based on 10), and the horizontal axis of the graph represents the evolutionary algebra of the algorithm.

**Figure 2:**
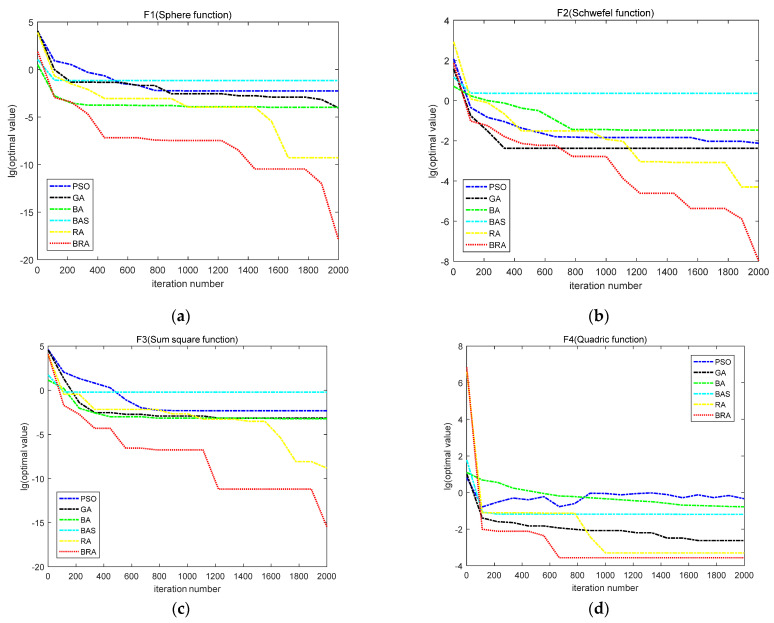

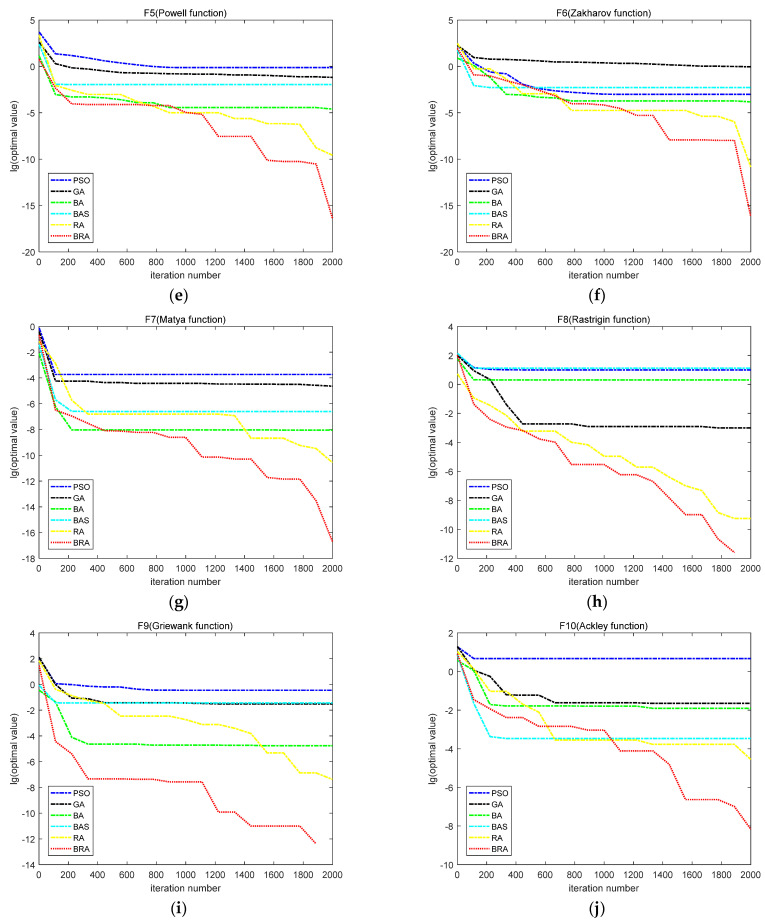
The convergence curves of 10 benchmark functions: (**a**) The convergence curves of F1; (**b**) The convergence curves of F2; (**c**) The convergence curves of F3; (**d**) The convergence curves of F4; (**e**) The convergence curves of F5; (**f**) The convergence curves of F6; (**g**) The convergence curves of F7; (**h**) The convergence curves of F8; (**i**) The convergence curves of F9; (**j**) The convergence curves of F10.

As can be seen from Figure 2, for the $F_{1}$, $F_{3}$, $F_{4}$, $F_{8}$ and $F_{9}$ functions, the BRA had the best convergence speed and solution accuracy compared with the other five comparison algorithms. The BRA can quickly reach the optimal value in the early stage, and then reach local optimization to improve the accuracy of the solution. For the $F_{2}$, $F_{5}$, $F_{6}$, $F_{7}$ and $F_{10}$ functions, the convergence speed of the BRA is not outstanding in the early stage, but the convergence speed and solution precision of the BRA in the later stage are improved greatly, which shows the excellent ability of the algorithm in balancing global and local optimization.

In a word, 10 classic function optimization problems were selected to test the optimization performance of the BRA. Compared with five well-known algorithms (PSO, GA, BA, BAS, RA), the results were analyzed statistically, and the hybrid strategy of the BAS algorithm and the RA presented in this paper can improve the performance of the two original algorithms.

## 5. Application Studies on Logistics Distribution Centers’ Location

In order to verify the effectiveness of the BRA for the location selection of the logistics distribution center, this study obtained the information of 31 urban geographic locations that need logistics distribution and selected Equation (1) as the objective function. The mathematical model of the logistics distribution centers’ location was established, and the BRA was compared with PSO, the GA, the BA, the BAS algorithm and the RA. A total of 31 cities were supposed to need one demand point each, and 6 were chosen as logistics distribution centers from 31 demand points. The location and demand of address are shown in Table 3.

**Figure 3:**
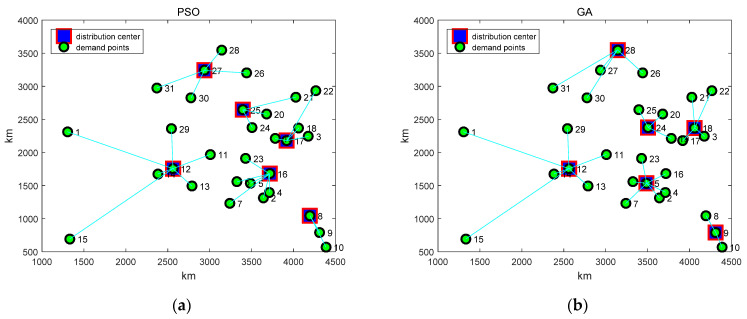

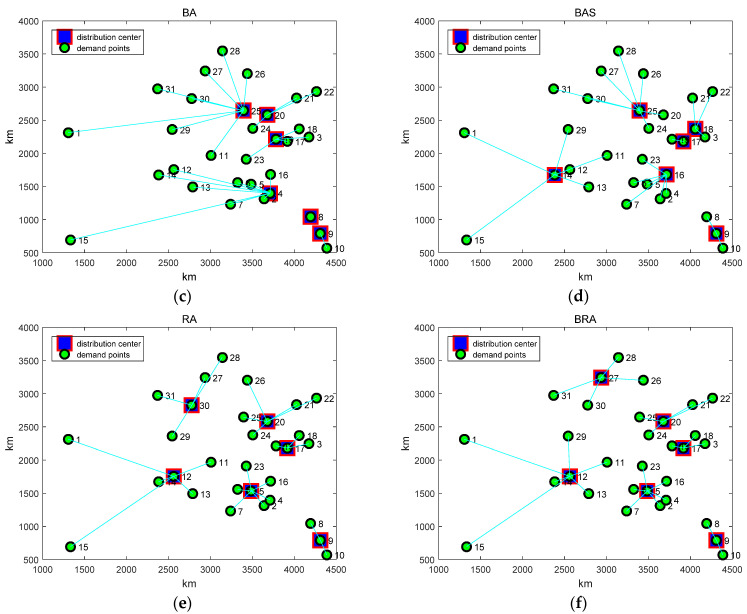
The selection schemes of the proposed BRA and other five comparison algorithms: (**a**) The selection scheme of PSO; (**b**) The selection scheme of GA; (**c**) The selection scheme of BA; (**d**) The selection scheme of BAS; (**e**) The selection scheme of RA; (**f**) The selection scheme of BRA.

Figure 3 shows six algorithms for the location of the logistics center, and the distribution center shown in the figure is the algorithm for the optimal solution. Taking Figure 3a as an example, distribution center 27 is responsible for the distribution tasks of demand points 26, 28, 30, 31, and distribution center 25 is responsible for the distribution tasks of demand points 20, 21, and 24. Distribution center 17 is responsible for providing services for demand points 3, 18, 19, 22, and distribution center 12 is responsible for delivery services for demand points 1, 11, 13, 14, 15, and 29. Distribution center 16 provides distribution services for demand points 2, 4, 5, 6, 7, 23, and distribution center 8 provides distribution services for demand points 9, 10. By analogy, Figure 3f shows the location scheme of the BRA, which is well understood. The box represents the distribution center, the dot represents the demand point, and the line between the box and the dot indicates that the goods at a demand point are distributed by the logistics distribution center. The BRA proposed in this paper is used to optimize the location model of the logistics distribution center, and the solution is [5,9,12,17,20,27].

**Figure 4:**
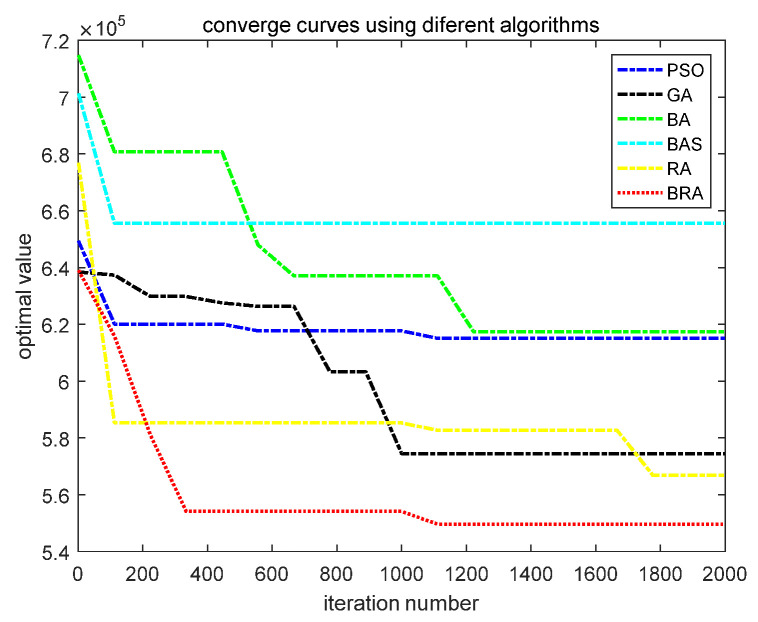
Convergence curve of six algorithms for the best result.

In order to compare the results of these six algorithms for the logistics distribution centers’ location problem more intuitively, the convergence curves of these six algorithms are shown in Figure 4. The vertical axis of the graph represents the optimal value of the objective function, and the horizontal axis represents the evolutionary algebra of the algorithm. As can be seen from Figure 4, compared with PSO, the GA, the BA, the BAS algorithm and the RA, the BRA has the best solution precision and optimization speed. The solution quality and convergence speed of the RA are second only to the BRA. The GA is inferior to the BRA and RA in accuracy and convergence speed, but superior to PSO and the BA. The BAS algorithm has the slowest searching speed and the lowest solution precision.

Table 4 shows the optimal results of six intelligent algorithms, PSO, GA, BA, BAS, RA and BRA, for the logistics distribution centers’ location problem.

From the statistical results in Table 4, it can be seen that, compared with the other five known algorithms, the BRA proposed in this paper has the smallest optimal results, that is, the lowest cost. The optimal fitness function value $f^{*}(\vec{x})=5.54\times 10^{5}$ is obtained at solution ${\vec{x}}^{*}=(5,27,9,20,12,17)$. The optimal value obtained by the RA is second only to the BRA, and a better fitness function value $f^{*}(\vec{x})=5.67\times 10^{5}$ is obtained at solution ${\vec{x}}^{*}=(5,9,30,20,12,17)$. The results of the GA are worse than those of the BRA and the RA, but better than those of PSO, the BA and the BAS algorithm, and the fitness function value $f^{*}(\vec{x})=5.74\times 10^{5}$ is obtained at solution ${\vec{x}}^{*}=(30,20,9,14,17,5)$. On the whole, the BRA is better than the other five contrast algorithms, and it has the lowest cost. The optimal values obtained by the RA and GA are slightly worse than those obtained by the BRA, and the cost is in the middle. The optimal values obtained by the BAS algorithm have the worst results, and the cost of obtaining is the highest.

To sum up, the BRA proposed in this paper is used to solve the location problem of logistics distribution centers, which further verifies the optimization performance of the BRA by comparing the calculated results with those of known algorithms PSO, GA, BA, BAS, and RA. The statistical analysis shows that the convergence speed and solution quality of the BRA are better than those of the contrast algorithm, and the BRA can be used to solve the location problem of logistics distribution centers, which can provide reference for actual logistics location planning.

## 6. Conclusions

A hybrid algorithm has been designed to solve the problem that the individual search in the BAS algorithm easily falls into the local optimum, as well as the slow convergence speed and the low searching precision. The hybrid algorithm embeds the BAS algorithm into the RA, which makes up the shortcomings of using the BAS algorithm for individual search and integrates the dynamic step strategy of the BAS algorithm into the RA effectively. The RA can effectively balance all and local search capabilities. Finally, by eliminating the inferior solution and generating the same number of solutions in the region near the global optimal solution, the diversity of the hybrid algorithm is improved, and the ability of the BRA to fine tune is enhanced, which reduces the possibility of the algorithm falling into the local optimum. The BRA was used to solve 10 function optimization problems, and the simulation results verified the effectiveness of the proposed algorithm. It was used to solve the location problem of logistics distribution centers, and the comparative analysis shows that the algorithm is an effective method to solve the location problem of logistics distribution centers. It can provide a new idea for decision makers to carry out scientific logistics network planning.

## Figures and Tables

**Table 1:** Benchmark functions.

No.	Name	Formula	D	Range	Optima
F1	Sphere	$F_{1}(x)=\sum_{i=1}^{D}x_{i}^{2}$	10	[−100, 100]	0
F2	Schwefel 2.22	$F_{2}(x)=\sum_{i=1}^{D}\left|x_{i}\right|+\prod_{i=1}^{D}\left|x_{i}\right|$	10	[−10, 10]	0
F3	Sum square	$F_{3}(x)=\sum_{i=1}^{D}ix_{i}^{2}$	10	[−10, 10]	0
F4	Quadric	$F_{4}(x)=\sum_{i=1}^{D}ix_{i}^{4}+\mathrm{random}(0,1)$	10	[−1.28, 1.28]	0
F5	Powell	$F_{5}(x)=\sum_{i=1}^{n/k}\left[{\left(x_{4i-3}+10x_{4i-2}\right)}^{2}+5{\left(x_{4i-1}-x_{4i}\right)}^{2}+{\left(x_{4i-2}-x_{4i-1}\right)}^{4}+10{\left(x_{4i-3}-x_{4i}\right)}^{4}\right]$	24	[−4, 5]	0
F6	Zakharov	$F_{6}(x)=\sum_{i=1}^{D}x_{i}^{2}+{\left(\sum_{i=1}^{D}0.5ix_{i}\right)}^{2}+{\left(\sum_{i=1}^{D}0.5ix_{i}\right)}^{4}$	10	[−5, 10]	0
F7	Matyas	$F_{7}(x)=0.26(x_{1}^{2}+x_{2}^{2})-0.48x_{1}x_{2}$	10	[−5, 10]	0
F8	Rastrigin	$F_{8}(x)=\sum_{i=1}^{D}(x_{i}^{2}-10\cos(2\pi x_{i})+10)$	10	[−5.12, 5.12]	0
F9	Griewank	$F_{9}(x)=\frac{1}{4000}\sum_{i=1}^{D}x_{i}^{2}-\prod_{i=1}^{n}\cos(\frac{x_{i}}{\sqrt{i}})+1$	10	[−600, 600]	0
F10	Ackley	$F_{10}(x)=-20\exp\left(-0.2\sqrt{\frac{1}{n}\sum_{i=1}^{n}x_{i}^{2}}\right)-\exp\left(\frac{1}{n}\sum_{i=1}^{n}\cos(2\pi x_{i})\right)+20+\exp(1)$	10	[−32, 32]	0

**Table 2:** Experimental results of benchmark functions.

**Function Criteria**	**F1 Sphere**				**F2 Schwefel 2.2.2**			
	**Best**	**Worst**	**Mean**	**Std**	**Best**	**Worst**	**Mean**	**Std**
PSO	5.14 × 10^−6^	1.23 × 10^−2^	1.50 × 10^−3^	2.20 × 10^−3^	4.50 × 10^−3^	1.206 × 10^−1^	2.87 × 10^−2^	2.52 × 10^−2^
GA	9.64 × 10^−5^	9.20 × 10^−3^	1.40 × 10^−3^	1.60 × 10^−3^	3.32 × 10^−4^	1.11 × 10^−2^	2.70 × 10^−3^	2.30 × 10^−3^
BA	6.38 × 10^−5^	1.87 × 10^−4^	1.18 × 10^−4^	3.91 × 10^−5^	2.53 × 10^−2^	3.43 × 10^−2^	2.91 × 10^−2^	3.43 × 10^−3^
BAS	6.79 × 10^−2^	4.69 × 10^−1^	3.16 × 10^−1^	1.49 × 10^−1^	6.39 × 10^−1^	2.84	1.47	7.11 × 10^−1^
RA	2.08 × 10^−8^	2.44 ×10^−10^	3.57 × 10^−9^	6.55 × 10^−9^	2.28 × 10^−5^	6.75 × 10^−7^	6.55 × 10^−6^	6.26 × 10^−6^
BRA	**4.50 × 10^−20^**	**2.10 × 10^−17^**	**6.65 ×10^−18^**	**6.82 × 10^−18^**	**3.63 × 10^−10^**	**7.07 × 10^−9^**	**1.99 × 10^−9^**	**2.27 × 10^−9^**
**Function Criteria**	**F3 Sum square**				**F4 Quadric**			
	**Best**	**Worst**	**Mean**	**Std**	**Best**	**Worst**	**Mean**	**Std**
PSO	1.00 × 10^−4^	1.86 × 10^−1^	2.02 × 10^−2^	3.53 × 10^−2^	6.70 × 10^−3^	4.27 × 10^−2^	2.48 × 10^−2^	8.60 × 10^−3^
GA	1.58 × 10^−5^	4.80 × 10^−2^	7.20 × 10^−3^	1.09 × 10^−2^	9.00 × 10^−4^	5.60 × 10^−3^	2.80 × 10^−3^	1.10 × 10^−3^
BA	3.74 × 10^−4^	7.21 × 10^−4^	5.41 × 10^−4^	1.12 × 10^−4^	9.15 × 10^−2^	3.21 × 10^−1^	1.74 × 10^−1^	6.56 × 10^−2^
BAS	6.07 × 10^−1^	1.19 × 10^1^	5.99	5.39	6.35 × 10^−2^	8.72	1.86	3.07
RA	1.04 ×10^−10^	1.49 × 10^−7^	2.43 × 10^−8^	4.60 × 10^−8^	1.38 × 10^−3^	2.87 × 10^−5^	4.22 × 10^−4^	3.97 × 10^−4^
BRA	**1.60 × 10^−19^**	**1.71 × 10^−17^**	**6.15 × 10^−18^**	**5.35 × 10^−18^**	**6.35 × 10^−6^**	**5.27 × 10^−4^**	**1.23 × 10^−4^**	**1.60 × 10^−4^**
**Function Criteria**	**F5 Powell**				**F6 Zakharov**			
	**Best**	**Worst**	**Mean**	**Std**	**Best**	**Worst**	**Mean**	**Std**
PSO	2.28 × 10^−1^	1.56 × 10^2^	5.21	21.9	1.94 × 10^−5^	33.7	6.74 × 10^−1^	4.76
GA	4.80 × 10^−3^	7.83 × 10^−2^	4.18 × 10^−2^	2.02 × 10^−2^	9.48 × 10^−2^	1.91	6.35 × 10^−1^	4.38 × 10^−1^
BA	1.89 × 10^−5^	1.38 × 10^−4^	5.40 × 10^−5^	3.53 × 10^−5^	1.38 × 10^−4^	3.16 × 10^−4^	2.11 × 10^−4^	6.25 × 10^−5^
BAS	4.01 × 10^−3^	2.42 × 10^−1^	5.98 × 10^−2^	1.02 × 10^−1^	1.77 × 10^−3^	1.19	4.78 × 10^−1^	5.87 × 10^−1^
RA	2.02 ×10^−13^	1.44 × 10^−9^	4.19 ×10^−10^	5.06 ×10^−10^	1.45 ×10^−11^	2.52 × 10^−8^	6.19 × 10^−9^	9.51 × 10^−9^
BRA	**2.38 × 10^−20^**	**9.40 × 10^−17^**	**3.24 × 10^−17^**	**3.39 × 10^−17^**	**1.37 × 10^−17^**	**2.12× 10^−15^**	**6.06× 10^−16^**	**7.47 × 10 ^−16^**
**Function Criteria**	**F7 Matyas**				**F8 Rastrigin**			
	**Best**	**Worst**	**Mean**	**Std**	**Best**	**Worst**	**Mean**	**Std**
PSO	1.67 × 10^−2^	9.61	6.70	2.08	1.67 × 10^−2^	9.61	6.70	2.08
GA	1.13 × 10^−6^	5.50 × 10^−3^	7.29 × 10^−4^	9.82 × 10^−4^	1.13 × 10^−6^	5.50 × 10^−3^	7.29 × 10^−4^	9.82 × 10^−4^
BA	2.01	9.97	6.78	3.09	2.01	9.97	6.78	3.09
BAS	1.71 × 10^−9^	3.35 × 10^−5^	7.14 × 10^−6^	1.48 × 10^−5^	1.19 × 10^1^	4.01 × 10^1^	2.39 × 10^1^	8.36
RA	2.28 ×10^−17^	2.60 ×10^−11^	4.74 ×10^−12^	8.74 ×10^−12^	1.10 ×10^−12^	1.60 × 10^−9^	5.09 × 10^−10^	5.99 ×10^−10^
BRA	**1.14 × 10^−20^**	**1.81 × 10^−17^**	**3.65 × 10^−18^**	**5.87 × 10^−18^**	**0**	**0**	**0**	**0**
**Function Criteria**	**F9 Griewank**				**F10 Ackley**			
	**Best**	**Worst**	**Mean**	**Std**	**Best**	**Worst**	**Mean**	**Std**
PSO	3.82 × 10^−2^	3.91 × 10^−1^	1.54 × 10^−1^	8.49 × 10^−2^	2.69	4.72	3.43	6.01 × 10^−1^
GA	4.74 × 10^−4^	1.09 × 10^−1^	4.93 × 10^−2^	2.54 × 10^−2^	1.43 × 10^−3^	2.24 × 10^−2^	1.01 × 10^−2^	6.01 × 10^−3^
BA	8.72 × 10^−6^	1.71 × 10^−5^	1.25 × 10^−5^	3.12 × 10^−6^	1.24 × 10^−2^	3.02	1.93	8.64 × 10^−1^
BAS	3.29 × 10^−2^	2.31 × 10^−1^	1.26 × 10^−1^	8.58 × 10^−2^	3.39 × 10^−4^	2.93 × 10^−2^	2.28 × 10^−3^	1.53 × 10^−3^
RA	6.21 ×10^−11^	2.64 × 10^−7^	4.52 × 10^−8^	9.22 × 10^−8^	1.94 × 10^−7^	4.50 × 10^−5^	1.77 × 10^−5^	1.44 × 10^−5^
BRA	**0**	**0**	**0**	**0**	**3.56 × 10^−11^**	**6.91 × 10^−9^**	**1.90 × 10^−9^**	**1.99 ×10^−9^**

**Table 3:** The coordinates and capacity of each demand point.

*i*	(*U_i_*, *V_i_*)	*c_i_*	*i*	(*U_i_*, *V_i_*)	*c_i_*	*i*	(*U_i_*, *V_i_*)	*c_i_*
1	(1304, 2312)	20	12	(2562, 1756)	40	23	(3429, 1908)	80
2	(3639, 1315)	90	13	(2788, 1491)	40	24	(3507, 2376)	70
3	(4177, 2244)	90	14	(2381, 1676)	40	25	(3394, 2643)	80
4	(3712, 1399)	60	15	(1332, 695)	20	26	(3439, 3201)	40
5	(3488, 1535)	70	16	(3715, 1678)	80	27	(2935, 3240)	40
6	(3326, 1556)	70	17	(3918, 2179)	90	28	(3140, 3550)	60
7	(3238, 1229)	40	18	(4061, 2370)	70	29	(2545, 2357)	70
8	(4196, 1044)	90	19	(3780, 2212)	100	30	(2778, 2826)	50
9	(4312, 790)	90	20	(3676, 2578)	50	31	(2370, 2975)	30
10	(4386, 570)	70	21	(4029, 2838)	50			
11	(3007, 1970)	60	22	(4263, 2934)	50			

**Table 4:** Optimal results for minimization of the cost.

Algorithms	Optimal Distribution Center (*j*)						Cost
	*j*_1_	*j*_2_	*j*_3_	*j*_4_	*j*_5_	*j*_6_	
PSO	27	16	25	17	8	12	6.15 × 10^5^
GA	30	20	9	14	17	5	5.74 × 10^5^
BA	19	9	25	20	8	4	6.08 × 10^5^
BAS	16	25	9	18	14	17	6.56 × 10^5^
RA	5	30	9	20	12	17	5.67 × 10^5^
BRA	5	27	9	20	12	17	5.54 × 10^5^

## Data Availability

Not applicable.
